# On Ozena

**Published:** 1851-07

**Authors:** M. Max-Simon


### ARTICLE XI.

### *On Ozena* By M. Max-Simon.

M. Max-Simon, in relating a case of this disease, makes several interesting remarks. Sometimes, he observes, it arises from ulceration of the Schneiderean membrane; at others, a deformity of the organ, whether congenital or acquired, prevents the free discharge of the mucosities of the nose, while in some cases there are signs of simple chronic inflammation. Another class of cases, of which this is an example, arises from a morbid condition of the economy at large, inducing modification in the condition of the secretion: just as we meet with intolerable stinking secretion from the feet, in persons perfectly clean; a peculiar odor from the axilla, occurring in others besides the red haired, in whom it is normal: and an insupportable condition of the breath in certain menstruating women. In such cases as these, the most careful examination exhibits no marks of inflammation or ulceration. The ulceration, in fact, which is met with in syphilis or other diseases, is not the cause of this peculiar stench, which is different from the smell they produce. A delicate young lady, æt. 14, had been under M. Simon's care for several years, for well marked ozena. Repeated examinations exhibited nothing abnormal, the mucous membrane being pale and not thickened, and the mucus itself normal in appearance, though sometimes increased in quantity. All medical treatment, whether local or general, proved quite unavailing, so that at last all was abandoned, but extreme cleanliness, and attention to maintain the general health in as good a condition as possible. However, menstruation came on, under the influence of which her system became much stronger, and her health far better, and the fetid odor diminished, to entirely disappear sixteen or eighteen months after the menses had become established. Another proof of the constitutional character of the disease in this case, and of the inutility of local measures, is found in the fact that a blister having been applied to the girl's arm, the serum furnished precisely the same smell. Her mother, too, suffered from the same infirmity, although by excessive cleanliness she kept it somewhat concealed. Her nose was a flat one, but that of her daughter was well formed.

These well-marked cases must not be confounded with a mere temporary unusual odor of the nasal mucus. In some persons this is liable to become accumulated or suppressed for a longer or shorter time. In the case of a young man, in whom there is no congestion of the mucus membrane, thickened mucosities are blown out from time to time, having a distinct spermatic smell. A young girl, at times, exhales mucus having the characteristic smell of ovena, and then, without the slightest care on her part, this disappears.

When the disease depends upon a mere perverted action of mucus membrane, the nitrate of silver injections are very useful; but when it is a constitutional affection, all local applications are mere palliatives. The greatest cleanliness is essential, and scented waters should be frequently injected into the nose. Sauvages found advantage in some cases, from the setting up a substitutive action by the use of snuff.—*Bulletin de Therapeut. Brit. & For. Med. Rev.*

